# Suppressing nonsense—a surprising function for 5-azacytidine

**DOI:** 10.15252/emmm.201404569

**Published:** 2014-11-06

**Authors:** Ada Shao, Miles F Wilkinson

**Affiliations:** 1 Department of Reproductive Medicine, University of CaliforniaSan Diego, CA, USAE-mail: mfwilkinson@ucsd.eduDOI 10.15252/emmm.201404569 | Published online 6 November 2014

## Abstract

In this issue of *EMBO Molecular Medicine*, Bhuvanagiri *et al* report on a chemical means to convert molecular junk into gold. They identify a chemical inhibitor of a quality control pathway that is best known for its ability to clear cells of rubbish, but that in certain cases can be detrimental because it eliminates “useful” garbage. The chemical inhibitor identified by Bhuvanagiri *et al* perturbs Nonsense-Mediated RNA Decay (NMD), a RNA surveillance pathway that targets mRNAs harboring premature termination codons (PTCs) for degradation (Kervestin & Jacobson, 2012).

See also: **M Bhuvanagiri *et al*** (December 2014)

“A little nonsense now and then is relished by the wisest men.” These words are from Roald Dahl, a writer lauded for his stories that veered toward the ridiculous, but who managed to probe into the inner psyche of children and adults alike. Likewise, biology takes advantage of what would appear to be junk. A duplicated gene copy that has deteriorated (a pseudogene) sometimes finds new life as a regulator of gene expression. Non-coding regions within coding genes (introns) engender a variety of functions. Transposable elements transform themselves over evolutionary time from mediators of havoc (which they invoke as they jump into and destroy useful genomic loci) to bearers of novel functions, including regulatory elements for neighboring genes.

Bhuvanagiri *et al* (2014) have identified a means to allow mutant genes containing aberrant stop codons to become useful. Stop codons are recognized by the translation apparatus; normally this event leads to termination of translation and release of the encoded protein. However, when a stop codon is in a premature context, this assembles components of the NMD machinery in a manner that recruits RNA degradation enzymes to rapidly degrade the PTC-bearing mRNA. A common means to generate in-frame PTCs is nonsense and frameshift mutations, the class of mutations responsible for causing one-third of human genetic disease cases (Holbrook *et al*, 2004). PTCs are also generated by biosynthetic errors, including aberrant alternative splicing and incorporation of inappropriate nucleotides during transcription. Thus, NMD is a major pathway for reducing the level of aberrant mRNAs. However, normal mRNAs can also sometimes be degraded by NMD. This occurs when a normal stop codon is in a context that is recognized as “premature”, such as when the stop codon is followed by a long 3′ untranslated region or an intron. The ability of NMD to degrade such “endogenous NMD substrates” is regulated and likely to be of biological value (Karam *et al*, 2013).

When it comes to disease, NMD is a two-edged sword. NMD is a weapon worth having when it targets a mutant disease-causing gene. PTCs lead to the generation of truncated proteins, some of which have dominant-negative activity that, for example, antagonize the functional protein expressed from the wild-type allele (Fig1). By rapidly degrading the mRNA encoding such dominant-negative proteins, NMD protects cells from their deleterious effects and thereby reduces or eliminates the symptoms of disease. Classic examples of this are β-thalassemia, von Willebrand disease, and Marfan syndrome (Holbrook *et al*, 2004).

**Figure 1:**
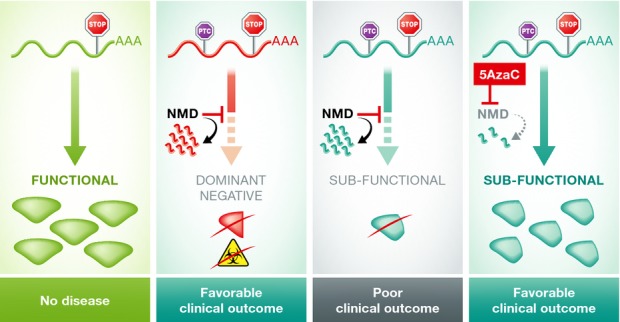
NMD inhibition therapy has the potential to improve the clinical outcome of a subset of human genetic diseases In cases where transcripts harboring a premature termination codon (PTC) produce a protein detrimental to the cell, NMD reduces the dominant-negative or toxic effects by targeting these transcripts for degradation. In transcripts in which the position of the PTC allows for the generation of a still functional protein, NMD is detrimental because it degrades the useful transcript. By reversing this decay, NMD inhibition therapy could improve disease symptoms.

While it serves as a useful RNA surveillance role in many circumstances, NMD can instead have a negative impact on cells. For example, if the gene mutation generates a PTC-containing transcript that produces a truncated protein retaining partial (or sometimes even complete) function, then degradation of the transcript by NMD will be counterproductive (Fig1). In the presence of NMD, less of the truncated functional protein will be produced, which can exacerbate disease symptoms. An example is cystic fibrosis patients who have severe disease when the mutation leads to the generation of a PTC-containing *CFTR* transcript encoding a still functional protein that is degraded by NMD (Holbrook *et al*, 2004). In such cases, NMD is a sword that turns on the bearer of the sword. Such patients would benefit from reduced NMD activity.

Currently known molecules that suppress NMD activity fall into one of the five following categories: NMD factor inhibitors, translation inhibitors, suppressor tRNAs, translation read-through inhibitors, and Ca^2+^ release inducers. Examples of the first class are wortmannin, caffeine, pateamine A, and NMDI-1, which inhibit different NMD components, including UPF1, SMG1, SMG7, and eiF4A3 (Keeling & Bedwell, 2011; Martin *et al*, 2014). Translation inhibitors suppress NMD because the recognition of the PTC depends on translation. Indeed, all known translation inhibitors block NMD, including cycloheximide, puromycin, anisomycin, and even viruses (Carter *et al*, 1995). Suppressor tRNAs repress NMD because they instruct the translation apparatus to interpret a stop codon as an amino acid-encoding codon, thereby suppressing translation termination. Likewise, read-through inhibitors increase the frequency at which ribosomes misinterpret stop codons, causing the ribosome to continue translation past the stop codon. Examples of read-through inhibitors include aminoglycosides (e.g., the antibiotics G418 and gentamycin) and the small molecule, PTC-124 (Keeling & Bedwell 2011). The final class of known NMD inhibitors is those that elevate intracellular Ca^2+^ levels (Nickless *et al*, 2014). Such inhibitors include the cardiac glycosides, ouabain, and digoxin. How increasing intracellular Ca^2+^ levels suppresses NMD is unknown.

Bhuvanagiri *et al* (2014) have identified a new NMD inhibitor to add to this arsenal: 5-azacytidine (5AzaC). This was a surprising finding, as 5AzaC is best known for its effects on DNA, not RNA. 5AzaC is a cytidine analogue that incorporates into DNA and inhibits DNA methyltransferases. Because 5AzaC blocks DNA methylation, cells treated with 5AzaC acquire hypomethylated DNA, leading to activation of genes previously suppressed by methylation, such as tumor suppressor genes. Given this activity, 5AzaC is used for the treatment of certain cancers; it has also proved to be effective for treating myelodysplastic syndrome patients. Bhuvanagiri *et al* (2014) identified 5AzaC in a screen to identify small molecules that could be rapidly applied in a therapeutic setting. Thus, they screened a library of drugs that had already been clinically licensed or were in advanced clinical development. Using a cell line expressing an NMD reporter, they screened 1,120 such clinically licensed drugs and identified several that inhibited NMD. One of these compounds—5AzaC—held up as a potent and specific NMD inhibitor at clinically relevant/therapeutic doses. Other hits identified in the screen, as well as chemical analogues of 5AzaC, either were only effective at doses too high for clinical use or had no effect on NMD.

Having found that 5AzaC is a potent NMD inhibitor, as judged by its effect on the levels of both an NMD reporter and endogenous NMD substrates, Bhuvanagiri *et al* (2014) next sought to determine the underlying mechanism of its action. They observed that 5AzaC increased the steady-state level of mature mRNA but not pre-mRNA from the NMD reporter, demonstrating that 5AzaC acts post-transcriptionally, as expected for a NMD inhibitor. They then tested various obvious mechanisms by which 5AzaC might inhibit NMD. Unfortunately, none of the mechanisms they tested were measurably affected by 5AzaC; it neither reduced the expression of known NMD factors, nor did it inhibit translational read-through or protein synthesis. Bhuvanagiri *et al* (2014) then elected to investigate the underlying mechanism using a proteomic approach. Using global mass spectrophotometry analyses, they found that 5AzaC upregulated 857 proteins and downregulated 1,002 proteins. One of the most strongly upregulated proteins was the oncoprotein, C-MYC. To test its causal role, the authors asked whether preventing C-MYC upregulation prevented 5AzaC's ability to inhibit NMD. Indeed, they found that C-MYC knockdown (to pre-treatment level) completely eliminated the anti-NMD activity of 5AzaC. This provided strong evidence that 5AzaC acts through C-MYC to inhibit NMD. Given that C-MYC is often overexpressed in tumors, one might expect that some tumors exhibit repressed NMD, which indeed has been shown to be the case (Wang *et al*, 2011b; Liu *et al*, 2014). Furthermore, it was previously shown that C-MYC overexpression is responsible for inhibiting NMD in tumors (Wang *et al*, 2011a).

The findings of Bhuvanagiri *et al* (2014) lead to an exciting new potential means to treat genetic diseases caused by mutant genes with nonsense or frameshift mutations that encode functional proteins. Because 5AzaC is already FDA approved and has been shown to have manageable side effects, it can potentially be rapidly repurposed into use for treating such “NMD-induced diseases”, such as Duchenne muscular dystrophy and cystic fibrosis. Indeed, 5AzaC is already approved for treatment of chronic diseases at doses that Bhuvanagiri *et al* (2014) found to effectively inhibit NMD. This means there will hopefully be few bureaucratic and clinical safety roadblocks to test its efficacy in patients.

While promising, there are also concerns with using 5AzaC therapeutically to treat genetic diseases. A potential one is that by inhibiting NMD, 5AzaC will promote the accumulation of PTC-containing transcripts that can cause toxicity or even new disease states. For example, by stabilizing PTC-bearing mRNAs encoding oncoproteins or dominant-negative tumor suppressors, 5AzaC therapy could promote the formation of tumors (Wang *et al*, 2011b; Liu *et al*, 2014). Another concern derives from the fact that NMD degrades not only aberrant transcripts, but also a subset of normal transcripts, some of which are important for normal developmental processes (Hwang & Maquat, 2011). While likely less of a concern in adults, where most developmental pathways are considered to be quiescent, treatment of children with an agent that significantly disrupts normal developmental processes could potentially be quite hazardous. Because of these potentially adverse consequences, the benefit-to-risk ratio of using 5AzaC must be carefully evaluated on a case-by-case basis. Nevertheless, this new NMD inhibitor offers promise as a course of treatment for patients suffering from the many diseases in which NMD either aggravates or produces a disease condition. Unleashing a little nonsense may indeed turn out to be a good thing.
